# Role of coronary arteries in patients with Acute Coronary Syndrome

**DOI:** 10.12669/pjms.39.6.7173

**Published:** 2023

**Authors:** Muhammad Usman, Hareem Zahra Qureshi, Kiran Zahid, Syed Naseem Bukhari

**Affiliations:** 1 Dr. Muhammad Usman, MBBS, FCPSI, MO, Department of Cardiology, Ch. Pervaiz Elahi Institute of Cardiology Multan, Multan - Pakistan; 2 Dr. Hareem Zahra Qureshi, MBBS, FCPSI, MO, Department of Cardiology, Ch. Pervaiz Elahi Institute of Cardiology Multan, Multan - Pakistan; 3 Dr. Kiran Zahid, MBBS, FCPSI, WMO, Department of Cardiology, Ch. Pervaiz Elahi Institute of Cardiology Multan, Multan - Pakistan; 4 Dr. Syed Naseem Bukhari, MBBS, FCPS, Assistant Professor, Department of Cardiology, Ch. Pervaiz Elahi Institute of Cardiology Multan, Multan - Pakistan

**Keywords:** Coronary arteries, Sudden cardiac arrest, Acute coronary syndrome, Coronary angiographic findings

## Abstract

**Objective:**

To assess the role of coronary arteries in acute coronary syndrome patients who have survived sudden cardiac arrest.

**Method:**

A cross-sectional study was conducted in Department of Cardiology at Chaudhary Pervaiz Elahi Institute of Cardiology, Multan from 1^st^ May 2021 to 1^st^ October 2021. A total of 203 patients who were diagnosed with sudden cardiac arrest were included as subjects of the study. Baseline data of all patients including age, sex, body mass index, history of smoking, diabetes and hypertension was noted. Coronary angiography was performed in all patients within five days after admission in hospital due to SCA.

**Results:**

The average age of patients was 52.61±11.09 years. There were 140 (68.97%) male and 63 (31.03%) female patients. There were 131 (64.53%) patients with STEMI and 72 (35.47%) patients with Non-STEMI. LMCAD were diagnosed in 29 (14.29%) patients, RCA in 88 (43.35%) patients, LAD in 174 (85.71%) Patients and LCX in 41 (20.20%) patients.

**Conclusion:**

LAD has the most involvement among the coronary arteries (85.71%) in patients with sudden cardiac arrest.

## INTRODUCTION

Despite of the advances in the acute medical care sudden cardiac arrest (SCA) carries the major burden of the mortality and morbidity in the emergency department worldwide and coronary artery disease (CAD) is the major cause of SCA.^1^,^2^ Most of these patients either have a ventricular fibrillation (VF) or pulseless ventricular tachycardia (PLVT)^3^ and more than 60% of these patients never achieve return of spontaneous circulation (ROSC).^4^

The “chain of survival” put a lot of emphasis on coronary angiography along with percutaneous coronary intervention (PCI) in the same setting when indicated which not only save the myocardium but also avoid recurrent arrest.^5^ Studies have concluded that early angiography and PCI are associated with reduced mortality rate in these patients and reduced risk of recurrent cardiac arrest.^6^,^7^ Davies et al reported 74% coronary thrombi causing death, and another study found 57% of cases showing acute coronary plaque on coronary angiography.^8^,^9^

There is a difference in published literature regarding the angiographic spectrum in patients with sudden cardiac arrest. However, no significant work has been done in Pakistan to evaluate the coronary artery involvement in SCA patients. The results of this study will help us to determine the common arteries causing SCA and the pattern of CAD in patients with SCA in our local population. The study aimed to assess the role of coronary arteries in acute coronary syndrome patients who have survived sudden cardiac arrest.

## METHODS

A cross-sectional study was conducted in the Cardiology department of Ch. Pervaiz Elahi Institute of Cardiology from 1^st^ May 2021 to 1^st^ October 2021. A total of 203 subjects were selected to be included in the study. Non-probability, consecutive sampling technique was used to select the patients. All the patients gave their written consent to become a part of the study.

### Ethical Approval

The ethical committee of the hospital approved by Ref# 15/29; dated 24-04-2021 the study design of the research.

SCA was defined as abrupt cessation of cardiac mechanical function, leading to death in the absence of reversal by a prompt intervention. Electrocardiograph (ECG) was used to diagnose cardiac arrest. The cardiac arrest was divided into two types, cardiac arrest ST elevation myocardial infarction (STEMI) which affects chambers of the heart mostly and due to Non-ST elevation myocardial infarction (NSTEMI) which is caused by insufficient oxygen supply.

STEMI was regarded as new ST elevation in at least two contiguous leads ≥ 1.5 mm in women or ≥ 2 mm in men in leads V2–V3 and/or of ≥ 1 mm in other contiguous chest or limb leads at J-point on ECG after reversion from cardiac arrest along with biochemical evidence (Cardiac troponin I >0.03 ng/ml) was labelled as having SCA due to STEMI. Patients having ST-depression (>1 mm) in limb or chest leads on ECG after reversion from cardiac arrest along with biochemical evidence (cardiac troponin I >0.03 ng/ml) was labelled as having SCA due to NSTEMI. Patients with prior myocardial infarction or those known cases of arrhythmias, diagnosed on previous medical records was excluded from the study. After including the patient in study and confirming the diagnosis of type of myocardial infarction and initial stabilization, patient’s coronary angiography was done within five days after admission in hospital due to SCA by consultant cardiologist or under the supervisor of consultant cardiologist.

The final angiography reporting was done by consultant cardiologist having a minimum five years post-fellowship experience. Presence of >70% stenosis in any of the three major epicardial coronary arteries was labelled as significantly diseased artery. Data regarding the patient’s age, gender, hypertension, diabetes, smoking, obesity, cause of SCA, and angiographic findings was collected.

### Statistical Analysis

All the data analyzed by SPSS version 20.0. Factors like age and body mass index were calculated in the form of mean and standard deviations. Whereas, smoking, obesity, gender, diabetes, angiographic findings and hypertension was calculated by percentage and frequency.

## RESULTS

A total 203 patients were included in the study. Mean age of patients included in this study was 52.61±11.09 years with age range from 30 to 70 years. Males (68.97%) were double the number of female population (31.03%). Mean body mass index (BMI) of patients was 24.87±3.35 kg/m^2^. The BMI range in subjects was 18.40 kg/m^2^ to 32.69 kg/m^2^. There were 72 (35.47%) smokers, 80 (39.41%) patients were diabetics, 108 (53.20%) were hypertensives and 77 (37.93%) were obese. There were 131 (64.53%) patients who suffered with cardiac arrest were having STEMI and 72 (35.47%) patients were diagnosed of having Non-STEMI. The involvement of different epicardial coronary arteries in patients of SCA on coronary angiography. Tale-I.

**Table-I:** Epicardial coronary artery involvement in patients of SCA on coronary angiography.

Coronary artery	Frequency	Percentage
Left main coronary artery	29	14.3%
Right coronary artery	88	43.3%
Left anterior descending artery	174	85.7%
Left circumflex artery	41	20.2%

## DISCUSSION

Sudden cardiac arrest can be caused due to multiple reasons and the disease progression is quite painful. Therefore, it is difficult to find out the cause and survival rate in patients even in this new era.^10^ In current study, we have evaluated the involvement of coronary arteries in SCA. The results showed that left anterior descending artery is most frequently involved in patients with SCA. 85.7% patients with SCA in our study had left anterior descending artery disease, a previous study reported this prevalence to be 79.5%.^11^

Sudden cardiac arrest is commonly caused by acute coronary syndrome (ACS). Diagnosing ACS is another challenge. Although, ST-segment elevation on ECG is commonly used for this purpose but it does not give accurate results and is neither specific for ACS, ST elevation can be caused by reasons other than acute coronary occlusion. In patients without ST-segment elevation, the diagnosis becomes more difficult.^6^ However, the process can be eased by percutaneous coronary intervention in early coronary angiography which improves its performance and survival rate in patients.^12^

A study conducted by Yannopoulos et al. on angiographic pattern of patients of SCA, the authors found Left main coronary artery disease (LMCAD) in 15% patients, left anterior descending (LAD) in 87%, left circumflex (LCx) in 52% and right coronary artery (RCA) disease in 50% patients of SCA.^13^ Another study by Redfors et al. on angiographic pattern of patients of SCA, found RCA in 22%, LMCAD in 5%, LAD in 52% and LCx in 20% patients.^14^ Our study also has comparable results. In present study, the most commonly involved artery was LAD followed by RCA and then LCx. Gracia et al^15^ showed that 35% of patients with sudden cardiac arrest have STEMI and Kern et al^16^ reported 27% of patients have STEMI. Our study showed that 64.53% patients have STEMI. This difference could be due to the fact that we emphasized on myocardial infarction as the cause of sudden cardiac death instead of including all other causes. But still our results are comparable as other studies still shows that major cause of SCA is STEMI. These findings will be help in screening and subsequent patients at increased risk of sudden cardiac arrest.

### Limitations

This was a descriptive observational, nonrandomized, single center study involving a small number of patients. Insufficient data was collected from patients in whom emergency angiogram was not performed. The frequency of acute myocardial infarction and coronary lesions in our study may be overestimated as it much lower in obvious non-ischemic and non-cardiac causes of SCA according to previous data.

## CONCLUSION

LAD has the most involvement among the coronary arteries (85.71%) in patients with sudden cardiac arrest.

### Authors Contribution:

**MU, HZQ and SNB:** conceived, designed and did statistical analysis & editing of manuscript

**KZ and MU:** did data collection and manuscript writing

**SNB:** did review and final approval of manuscript and also responsible for the integrity and accuracy of the study.
